# The role of the radiologist in the dengue endemic: a pictorial essay

**DOI:** 10.1590/0100-3984.2024.0077-en

**Published:** 2024-12-11

**Authors:** Edinaldo Gomes de Oliveira Neto, Danilo Delamare Lucena Nascimento, Tiago Vasques Bertoncini, Arthur Ataíde Lopes, Alexandre Sérgio de Araujo Bezerra, Mayra Veloso Ayrimoraes Soares

**Affiliations:** 1 Hospital Universitário de Brasília/Universidade de Brasília (HUB/UnB), Hospital Sírio-Libanês Brasília, Laboratório Exame/DASA, Brasília, DF, Brazil, Hospital Santa Marta (HSM), Taguatinga, DF, Brazil; 1

**Keywords:** Dengue, Diagnostic imaging, Emergencies, Dengue, Diagnóstico por imagem, Emergências

## Abstract

The dengue virus, a member of the family Flaviviridae, is transmitted by *Aedes* mosquitoes and causes a viral disease known as dengue fever that is prevalent in tropical and subtropical regions. It is estimated that there are 100–400 million new infections every year, with underreporting due to limited surveillance systems. The presentation ranges from asymptomatic to dengue shock syndrome. Brazil is now facing an endemic of dengue, having seen a significant seasonal increase of over 4.5 million in the number of probable cases reported. Imaging exams such as ultrasound, computed tomography, and magnetic resonance imaging are crucial for detecting complications of dengue, aiding in the clinical management and differential diagnosis, especially in severe cases. The aim of this study was to illustrate the radiological findings of dengue, focusing on emergency and critical care settings.

## Abstract (pt)

O vírus da dengue, membro da família Flaviviridae, causa uma arbovirose prevalente em regiões tropicais e subtropicais, transmitida por mosquitos *Aedes*. Estima-se entre 100–400 milhões de novas infecções anualmente, com subnotificação devidas a sistemas de vigilância limitados. A infecção pode variar de assintomática a síndrome do choque por dengue. Em 2024, o Brasil enfrenta uma situação endêmica de dengue, observando um aumento sazonal significativo com a notificação de mais de 4,5 milhões de casos prováveis. Exames de imagem como ultrassonografia, tomografia computadorizada e ressonância magnética são cruciais para detectar complicações, auxiliando no manejo clínico e no diagnóstico diferencial, especialmente em casos graves. O objetivo deste ensaio iconográfico é ilustrar os achados radiológicos da dengue, com ênfase no cenário de urgência e emergência.

## INTRODUCTION

The dengue virus is a member of the family Flaviviridae that affects many individuals living in tropical and sub-tropical regions**^(1)^**. Humans are typically infected with this arbovirus through bites from mosquitoes, especially those of the genus *Aedes*. It is estimated that the annual number of new dengue virus infections is between 100 million and 400 million worldwide. However, because most tropical countries do not have robust surveillance systems, it is likely that the number of cases is underreported**^(2)^**.

Dengue virus infection can cause a wide range of clinical symptoms, ranging from an asymptomatic phase to dengue shock syndrome**^(3)^**. The recent exponential in-crease in the prevalence of dengue puts almost half of the global population at risk. Despite being endemic in Brazil, dengue had low circulation in some states, especially in the southern and central-west regions, until recently, when those regions began to see significant seasonal increases in the incidence of the disease**^(4,5,6,7)^**. In the first months of 2024, Brazil showed a significant increase in the number of cases of dengue, which was addressed with public health measures and a national vaccination plan**^(8,9)^**, although more than 4.5 million probable cases were still reported. Approximately 80% of individuals infected with the dengue virus do not develop symptoms. When clinical manifestations are present, the disease can be classified, according to a 2009 publication by the World Health Organization**^(10,11)^**, as follows (Table 1): classic dengue; dengue with warning signs; and severe dengue.

**Table 1:** Clinical and radiological manifestations of dengue, by type (severity). (Modified from the 2009 World Health Organization classification^(10,11)^.

Type	Clinical manifestations	Radiological manifestations
Classic dengue	Fever + two other symptoms: • Skin rash • Vomiting/nausea • Myalgia • Leukopenia	None
Dengue with warning signs	Fever + any of the warning signs: • Abdominal pain • Persisting vomiting • Mucosa bleeding • Fluid accumulation • Increased hematocrit and thrombocytopenia • Lethargy • Hepatomegaly	Pericardial effusion Pleural effusion Ascites Hepatosplenomegaly Encephalitis Myelitis Pericarditis Bleeding Pancreatitis Cholecystitis Thickening of intra-abdominal fat
Severe dengue	Signs of shock Severe bleeding Organ dysfunction	Signs of shock

Examinations such as ultrasound, computed tomography (CT), and magnetic resonance imaging (MRI) facilitate the recognition of complications of dengue and, in select cases, even their diagnosis. Among these three examinations, ultrasound stands out for its accessibility, low cost, and portability**^(12,13)^**. The main radiological findings in severe dengue are thickening of the gallbladder wall, ascites, pleural effusion, hepatomegaly, and splenomegaly. However, those findings are not specific or pathognomonic and can be associated with several diseases, one example being diffuse thickening of the gallbladder wall, which could also be attributed to acalculous cholecystitis**^(14)^**. In this scenario, the radiologist plays an important role. Through the use of imaging methods, radiologists aid in the investigation of signs of severity, in the monitoring of complications, and in the differential diagnoses.

## RADIOLOGICAL MANIFESTATIONS

The main pathophysiological mechanism of severe dengue is platelet destruction and consumption, together with increased vascular permeability, resulting in polyserositis**^(10)^**. That explains, in part, the findings of diffuse gallbladder wall thickening, ascites, hepatosplenomegaly, and pancreatitis. Unusual complications such as spontaneous abdominal hemorrhage, especially retroperitoneal hemorrhage, can also occur in patients with severe thrombocytopenia.

**Figure 1:**
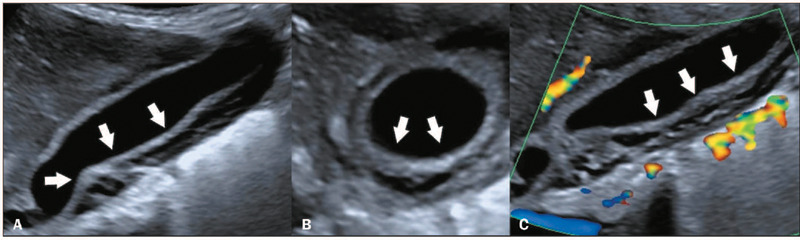
Diffuse thickening of the gallbladder in a six-year-old female patient diagnosed with dengue. Doppler ultrasound in the sagittal, axial, and sagittal planes (**A, B,** and **C,** respectively), showing diffuse thickening of the gallbladder wall (white arrows), with a “honeycomb” pattern.

**Figure 2:**
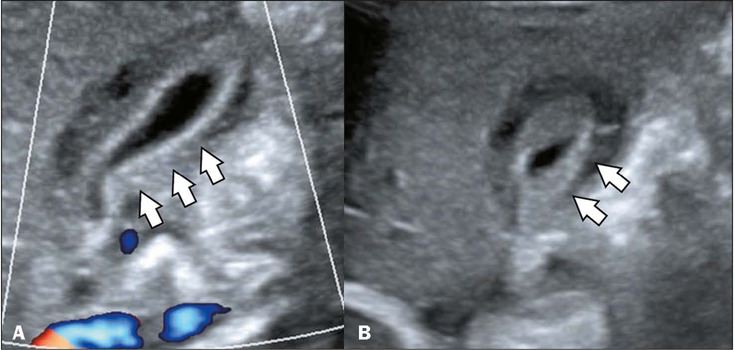
Diffuse thickening of the gallbladder in a nine-month-old female patient diagnosed with dengue. Doppler ultrasound in the sagittal and axial planes (**A** and **B**, respectively), showing diffuse thickening of the gallbladder wall, with a “honeycomb” pattern (arrows)).

**Figure 3:**
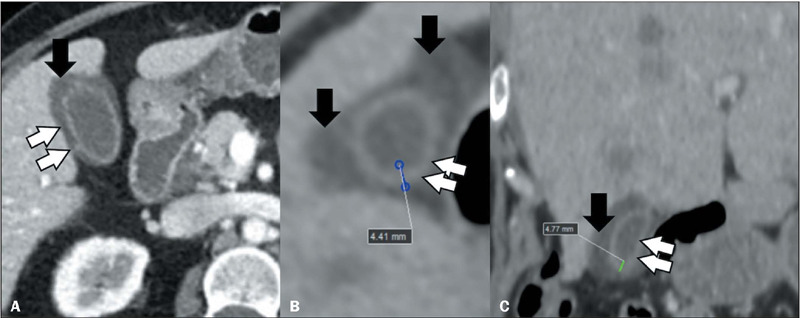
Diffuse gallbladder thickening in a patient with abdominal pain and a diagnosis of dengue. Contrast-enhanced CT in the axial plane **(A)** showing diffuse thickening of the gallbladder wall (white arrows) and free pericholecystic fluid (black arrows). Unenhanced CT in the axial and coronal planes (**B** and **C**, respectively), also showing diffuse thickening of the gallbladder wall (white arrows), and free pericholecystic fluid (black arrows).

The gallbladder is an elongated organ with a folded fundus, and its primary function is to be a reservoir for bile synthesized at the hepatocyte level. Its thickening can be caused by inflammatory, benign, or malignant processes and is defined as a wall thickness greater than 3 mm**^(13)^**. In cases of dengue, that finding could lead to confusion with other diagnoses, such as acalculous cholecystitis. On ultrasound, gallbladder thickening presents as one of four patterns**^(15)^**: uniform echogenic; striated with multiple hypoechoic layers with echogenic zones between them; a central hypoechoic layer separated by two echogenic layers; and asymmetric with projection of echogenic tissue into the lumen. In cases of severe dengue, the “honey-comb” pattern (Figures 1, 2, and 3) has high sensitivity and specificity when accompanied by ascites, pleural effusion, hepatomegaly, or splenomegaly**^(16)^**.

**Figure 4:**
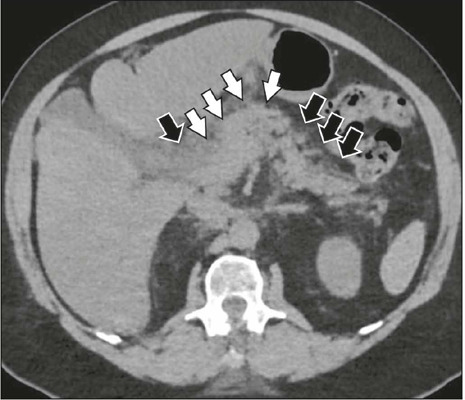
Pancreatitis in a patient with a confirmed diagnosis of dengue. Unenhanced CT in the axial plane, showing thickening of the pancreatic body (white arrows) and infiltration of the peripancreatic fat (black arrows).

**Figure 5:**
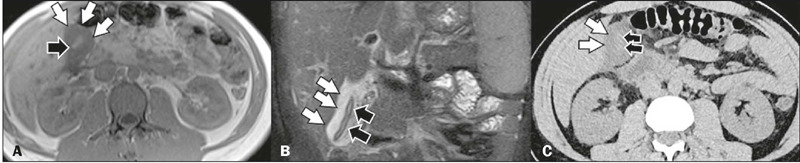
Bleeding in the internal cavity of the gallbladder in a patient diagnosed with dengue. Axial T1-weighted MRI **(A)** showing the gallbladder with diffuse wall thickening (white arrows) and contents with a diffuse hyperintense signal (black arrow). Coronal T2-weighted MRI with fat suppression (**B**), showing the gallbladder with diffuse wall thickening (white arrows) and contents with a hyperintense signal (black arrows). Axial unenhanced CT (**C**), also showing the gallbladder with diffuse wall thickening (white arrows) and hyperdense contents (black arrows).

The processes associated with pancreatitis and chole-cystitis after dengue infection are not yet fully understood. One hypothesis is that direct viral invasion triggers a series of processes that prevent the outflow of pancreatic fluid, resulting in biliary stasis. Those processes include local inflammation, tissue edema, and the death of pancreatic acinar and gallbladder cells. Systemic inflammatory responses, secondary bacterial translocation, spasms of the ampulla of Vater, and ischemic lesions are other potential risk factors for acute cholecystitis. Acute pancreatitis (Figure 4) can be caused by an autoimmune response to pancreatic islet cells, provoked by the infection. The imaging manifestations of pancreatic involvement in dengue are indistinguishable from those seen in other viral diseases. The authors of one epidemiological study concluded that there is a significantly increased risk of acute cholecystitis and pancreatitis during the acute phase of infection with the dengue virus**^(14)^**. The complication of such an inflammatory process in the gallbladder is necrosis of its mucosa, with consequent intracavitary bleeding, as can be seen on MRI and CT (Figure 5).

**Figure 6:**
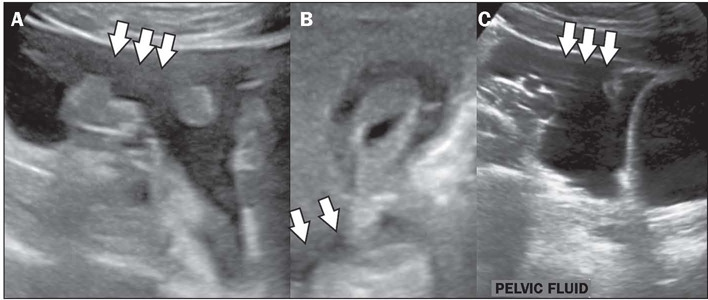
Ascites in a patient with abdominal pain and a confirmed diagnosis of dengue. Ultrasound in the sagittal plane (**A**), in the axial plane (**B**), and again in the sagittal plane (**C**), showing a small to moderate amount of free fluid in the abdominal cavity (arrows).

**Figure 7:**
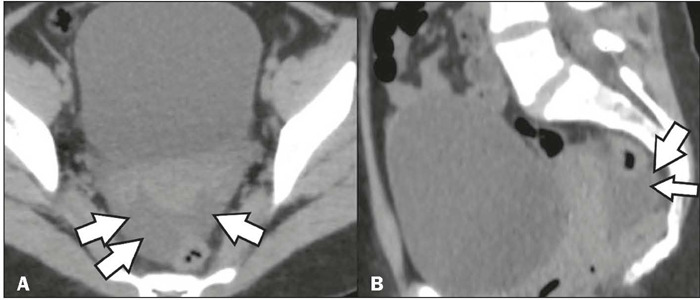
Ascites in a patient with abdominal pain and a confirmed diagnosis of dengue. Unenhanced CT in the axial and sagittal planes (**A** and **B**, respectively), showing a small to moderate amount of free fluid in the abdominal cavity (arrows).

Ascites (Figures 6 and 7) is defined as the accumulation of fluid of pathological origin in the abdominal cavity**^(17)^**. Patients with a large volume of ascites can present abdominal distension (which can be painful), nausea, vomiting, dyspnea, and peripheral edema. This condition has a wide range of etiologies and is a common finding in severe dengue. Its pathophysiological mechanism in dengue is based on an anomalous immune response, promoting increased vascular permeability, resulting from dysfunction of the vascular endothelium. Consequently, there is interstitial extravasation of fluid, which lowers blood pressure, and thrombocytopenia, which results in hemorrhagic manifestations**^(18)^**. Ascites can be related to changes in the liver parenchyma and pleural effusion, accompanied by intraparenchymal and subcapsular bleeding in some cases**^(12)^**.

**Figure 8:**
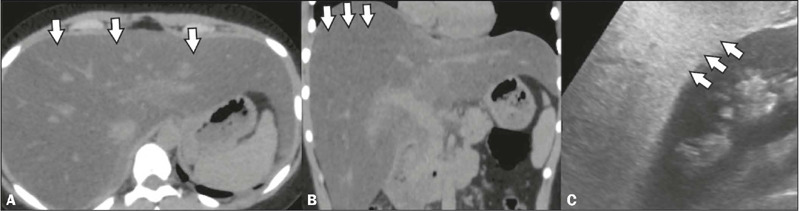
Hepatomegaly in a patient with a confirmed diagnosis of dengue. Unenhanced CT in the axial and coronal planes (**A** and **B**, respectively), showing hepatomegaly and diffuse hypoattenuation of the liver parenchyma (arrows). Ultrasound in the sagittal plane (**C**), also showing hepatomegaly (arrows).

**Figure 9:**
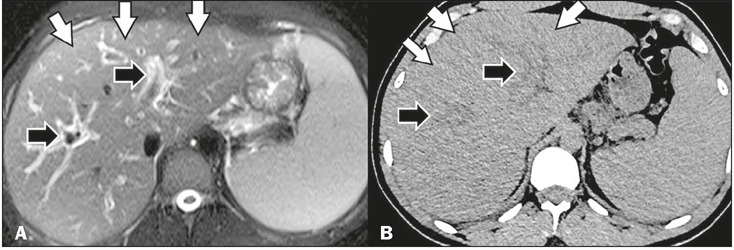
Hepatomegaly and periportal edema in a patient with a confirmed diagnosis of dengue. T2-weighted MRI with fat suppression (**A**) and unenhanced CT in the axial plane (**B**), showing hepatomegaly (white arrows) and mild periportal edema (black arrows).

**Figure 10:**
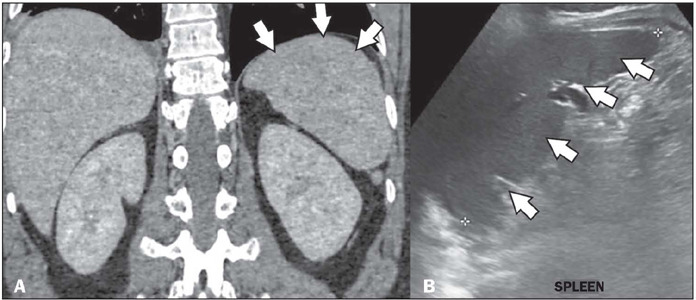
Splenomegaly in a patient with a confirmed diagnosis of dengue. Unenhanced CT in the coronal plane (**A**), showing splenomegaly. Ultrasound in the sagittal plane (**B**), also showing splenomegaly (arrows).

The pathophysiology of liver lesions in dengue is directly related to hepatocyte apoptosis caused by the virus, hepatitis induced by immune-mediated hepatocyte lesions, and a cytokine storm**^(19)^**. Hepatomegaly (Figures 8 and 9) is a common finding in severe dengue and is commonly accompanied by splenomegaly**^(20)^**, as illustrated on CT in Figure 10. In an autopsy study**^(21)^**, 58% of the cases of dengue presented hepatomegaly associated with parenchymal alterations (Figure 9), such as steatosis, focal necrosis, and hemorrhage, findings that are not pathognomonic and can be observed in other viral diseases. Splenic congestion and subcapsular hematomas were found in 15% of the cases.

**Figure 11:**
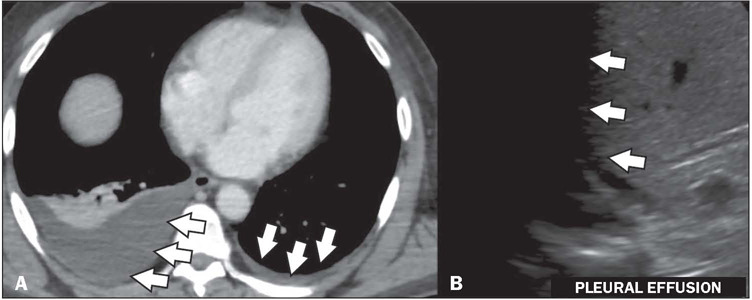
Pleural effusion in a patient with a confirmed diagnosis of dengue. Contrast-enhanced axial CT in the portal phase (**A**), showing bilateral pleural effusion, small to moderate on the right and laminar on the left showing a small pleural effusion (arrows).

**Figure 12:**
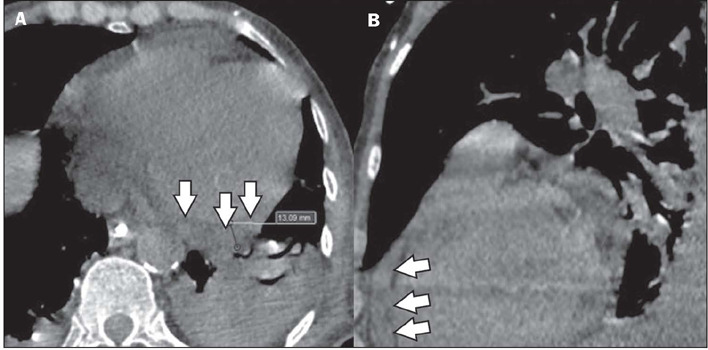
Pericardial effusion in a patient with a confirmed diagnosis of dengue. Unenhanced CT in the axial and sagittal planes (**A** and **B**, respectively), showing a small pericardial effusion (arrows) with a maximum thickness of 13 mm.

**Figure 13:**
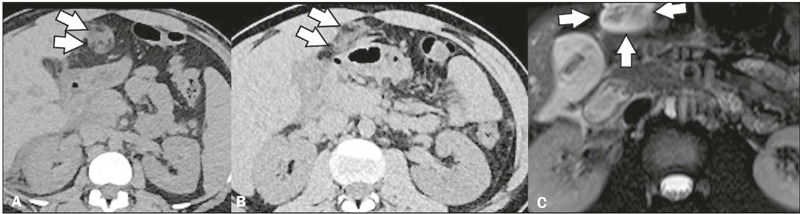
Infiltration of the fat planes adjacent to the round ligament of the liver in a patient with a confirmed diagnosis of dengue. Unenhanced CT in the axial planes (**A, B**) and axial T2-weighted MRI with fat suppression (**C**), showing densification of the fat planes adjacent to the round ligament of the liver (arrows).

Fluid accumulation in the cavities, resulting from increased capillary permeability, can cause additional unusual abdominal and extra-abdominal findings, such as periportal edema (Figure 9), pleural effusion (Figure 11), pericardial effusion (Figure 12), and thickening of the ligamentum teres, or round ligament of the liver (Figure 13). Early identification of these signs is important for effective therapeutic practice. Although point-of-care ultrasound has the potential to be an important tool in the emergency setting, no protocols for its use have yet been established**^(22)^**. In 2024, the first structured ultrasound protocol for evaluating dengue-related complications was described, aimed at the emergency department, which could increase the efficacy of care of patients with more severe disease**^(23)^**.

**Figure 14:**
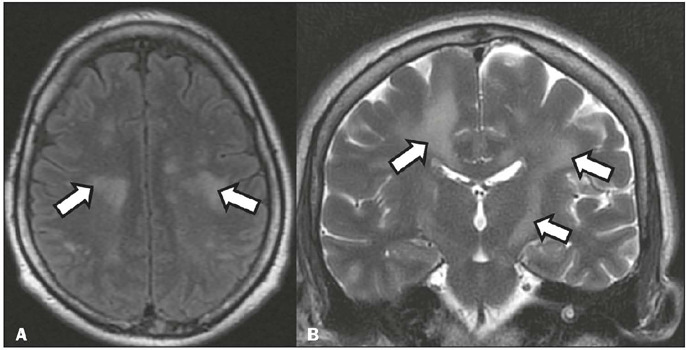
A 52-year-old patient with decreased consciousness and left hemiparesis, consistent with dengue-induced encephalitis. Axial T2-weighted MRI with cerebrospinal fluid saturation (**A**) and coronal T2-weighted MRI (**B**), showing white matter lesions (arrows), consistent with dengue-induced encephalitis. (Images kindly provided by Dr. Amina Muhamad Mota Mustafá and Dr. Alexsandra Rossi).

**Figure 15:**
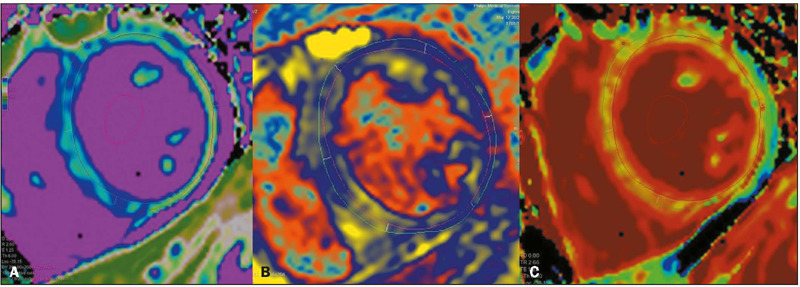
A 29-year-old patient with acute dengue-induced myocarditis. MRI with native T1 mapping of myocardial tissue (**A**), T2 mapping of myocardial tissue (**B**), and depiction of myocardial extracellular volume (**C**), showing diffuse signal alteration, indicating a myocardial inflammatory process. (Images kindly provided by Dr. Joalbo Matos de Andrade)..

Neurological (central nervous system) manifestations are rare and are generally divided into encephalopathy, related to systemic conditions; encephalitis (Figure 14), caused by direct invasion of the virus; and immune-mediated demyelination or vasculitis. The changes caused by encephalitis can be observed on MRI and are characterized by areas of bilateral hyperintense signal on T2-weighted MRI sequences**^(24)^**, as shown in Figure 14. Although rare, cardiac involvement can occur in dengue. The dysregulated inflammatory response to the virus can affect the cardio-vascular system, causing symptoms that range from mild, such as palpitations and dyspnea, to severe, such as cardio-genic shock, acute heart failure, arrhythmias, pericarditis, and myocarditis**^(25)^**, as depicted on MRI (Figure 15).

## CONCLUSION

There is a broad spectrum of manifestations of dengue and its complications on imaging. Radiological imaging methods are extremely important, especially in recognizing complications related to the disease and monitoring the affected patients. It is essential that the radiologist quickly identifies signs of severity, assessing the complications and possible differential diagnoses of this disease that is endemic in Brazil.
